# Re-recognize early recurrence of persistent atrial fibrillation

**DOI:** 10.3389/fcvm.2023.1145695

**Published:** 2023-05-30

**Authors:** Kaixuan Fu, Xuefeng Zhu, Hongxia Chu, Lin Zhong, Zhen Wang, Wenjing Li, Chunxiao Wang, Jianping Li, Lei Gong, Guangqiang Wang, Rao Yao, Lihong Wang

**Affiliations:** ^1^ Department of Cardiology, The Affiliated Yantai Yuhuangding Hospital of Qingdao University, Yantai, China; ^2^ Doppler Ultrasonic Department, The Affiliated Yantai Yuhuangding Hospital of Qingdao University, Yantai, China

**Keywords:** persistent atrial fibrillation, blanking period, catheter ablation, early recurrence, late recurrence

## Abstract

**Aims:**

Few studies on early recurrence (ER) focused on patients with persistent atrial fibrillation (AF). We aimed to investigate the characteristics and clinical significance of ER in patients with persistent AF after catheter ablation (CA).

**Methods:**

A total of 348 consecutive patients who underwent first-time CA for persistent and long-standing persistent AF between January 2019 and May 2022 were investigated.

**Results:**

About 5/348 (1.44%) patients who failed to convert to sinus rhythm after CA were excluded. A total of 110/343 (32.1%) patients had ER, in which 98 (89.1%) were persistent and 50.9% occurred in the first 24 h after CA. Compared with the patients without ER, those with ER were more likely to have late recurrence (LR) (92.7% vs. 1.7%, *P* < 0.001) during a median follow-up of 13 (IQR 6–23) months. ER was the most significant independent predictor for LR (OR 120.5, 95% CI 41.5–349.8, *P* < 0.001). ER as atrial flutter (AFL) had a lower risk of LR when compared with ER as AF (*P* = 0.011) and both AF and AFL (*P* = 0.003). Early intervention of the patient with ER improved the short-term outcomes (*P* < 0.001), not long-term outcomes. Only 22/251 (8.76%) patients of LR appears among those who had no recurrence in the first month.

**Conclusions:**

Patients with persistent AF may not have a blanking period but rather have a risk period. Clinical significance of the blanking period should be given differential treatment between paroxysmal AF and persistent AF.

## Introduction

Catheter ablation (CA) is used to treat symptomatic atrial fibrillation (AF), but recurrences are common after initially successful CA (1). Early recurrence (ER) occurs during the first 3 months after CA, the so-called “blanking period,” is estimated to be as high as 50% (2–4), and is not considered to be a failure of AF ablation (5, 6) also not suggesting reintervention.

However, patients with ER are at higher risk to develop late recurrence (LR) (3, 5, 7). ER is more common in the patients with persistent AF undergoing CA than in those with paroxysmal AF (5, 6). Previous studies on ER in AF mostly focused on patients with paroxysmal AF (8). The differences in ER between patients with persistent AF and those with paroxysmal AF have not been sufficiently identified. The current study sought to characterize ER in patients with persistent AF after CA during the blanking period and to determine its prognostic significance.

## Methods

### Study population

A total of 348 patients undergoing the first CA for drug-refractory persistent or long-standing persistent AF in the Yantai Yuhuangding Hospital from January 2019 to May 2022 were enrolled. The type of AF was defined according to generally accepted guidelines (1). All patients provided written informed consent for the ablation procedure and the use of their clinical data for this retrospective study. This study was approved by the Ethics Committee of our institution.

### Ablation procedure

**Figure 1:**
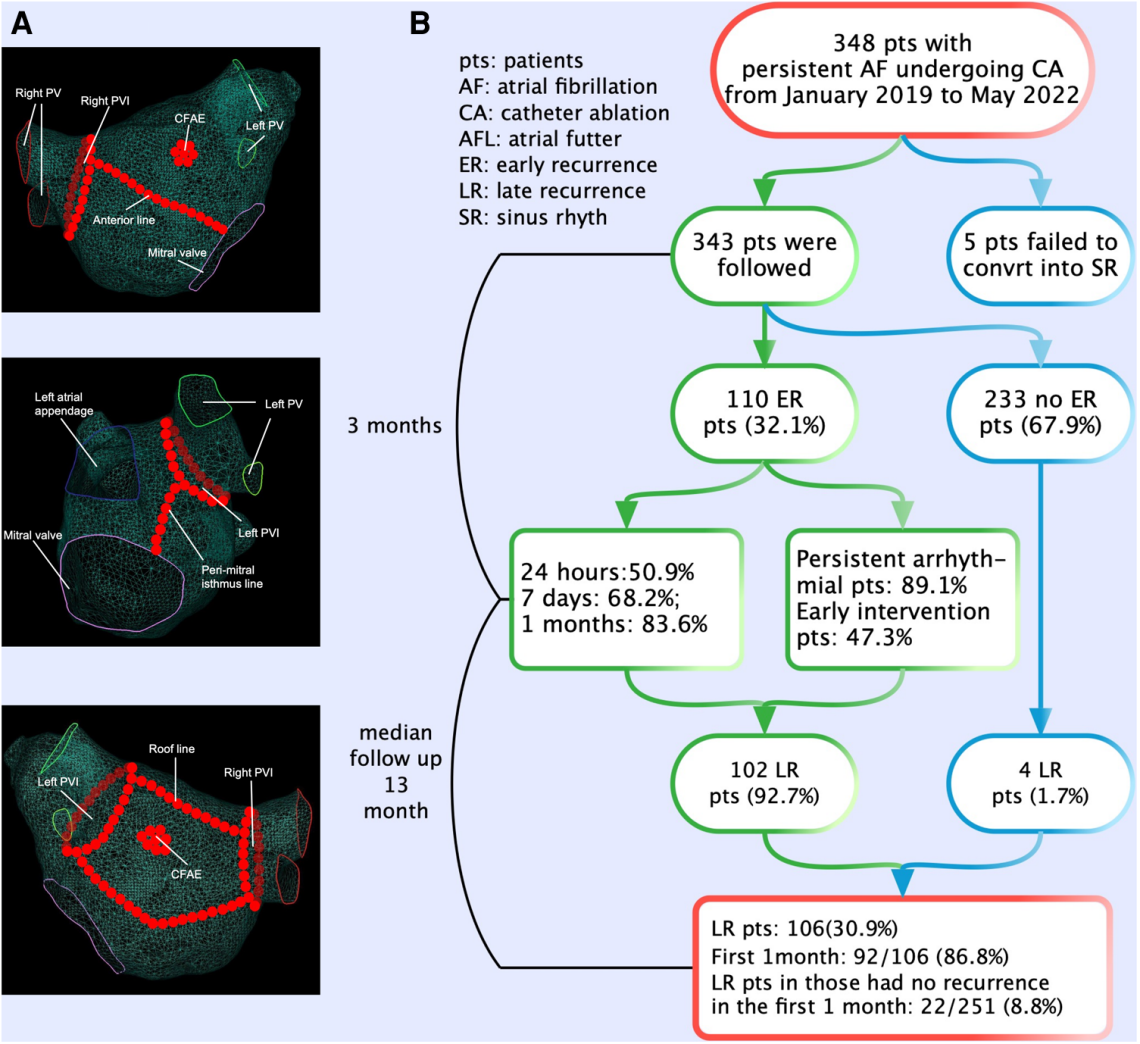
The ablation strategies and flowchart of the present study. (**A**) All patients underwent extensive encircling pulmonary vein isolation. Additional substrate ablations (such as linear ablation, low-voltage zone ablation, or complex fractionated atrial electrogram ablation) depended on the operator's judgment. (**B**) The flowchart of the study. A total of 348 patients with persistent AF underwent CA. Patients experiencing ER have a significantly higher risk (92.7%) of LR compared with those without ER. The majority (50.9%) of ER first occurred in the first 24 h after CA. ER was predominantly present as persistent. AF, atrial fibrillation; CA, catheter ablation; ER, early recurrence; LR, late recurrence.

Details of the periprocedural management and catheter placement has been published previously (9). All procedures were guided by the CARTO 3 (Biosense Webster) electroanatomic mapping system, and ablation was performed using open irrigated catheters with contact force (CF) sensing (Thermocool Smart Touch, Biosense Webster). Ablation index (AI) was introduced as a novel algorithm of ablation lesion quality evaluation based on parameters that were formed by contact force-time power measured by the CF-sensing catheter. The AI could reliably predict the degree of necrosis in RF delivery. All patients underwent extensive encircling pulmonary vein isolation (PVI). Other additional substrate ablations [such as linear ablation, low-voltage zone ablation, or complex fractionated atrial electrogram (CFAE) ablation] (Figure 1A) depended on the operator's judgment. The endpoint of the linear ablation was a complete, bidirectional block across the linear lesion. If sinus rhythm (SR) was not transformed after ablation procedures, it was restored by Ibutilide. If it still failed, cardioversion was performed. All patients eventually returned to SR and were observed for 30 min without recurrence of arrhythmia. We selected previous studies about ER of AF as the control to research the difference in the characteristics and clinical significances of ER between persistent AF and paroxysmal AF.

### Follow-up

Patients underwent continuous inpatient electrocardiogram (ECG) monitoring during the first 48 h after CA. After discharge, oral anticoagulation (OA) was continued for at least 3 months after CA. Further use of OA was determined according to the ESC guidelines (1). Regardless of the presence or absence of ER, antiarrhythmic drugs were discontinued 3 months after CA. Follow-up was scheduled 1, 2, 3, 6, and 12 months after CA in our outpatient clinic and comprised clinical assessment. At all follow-up visits, 12-lead ECG were obtained and 24-h Holter monitoring was performed at 3-month intervals during the follow-up period. Patients were strongly encouraged to obtain an ECG and advised to carry a portable event recorder if any arrhythmic symptom occurred.

ER and LR were defined as any atrial tachyarrhythmia recurrence occurring ≥30 s after CA ≤90 and >90 days after CA, respectively. Recurrence of arrhythmia consisted of both AF and atrial flutter (AFL). The manifestation of ER and LR can be divided into paroxysmal and persistent according to whether it can transform into SR within 7 days after recurrence. ER events were then categorized into the following time periods: (i) very early ER (first 24 h after CA), (ii) early ER (first 24 h–1 week after CA), (iii) intermediate ER (1 week–1 month after CA); and (iv) late ER (1–3 months after CA).

### Intervention for ER

For patients with paroxysmal ER, intervention is not carried out, and for patients with persistent ER, early intervention (cardioversion and/or in combination with anti-arrhythmic drugs (AADs)) was performed voluntarily during the blanking period. If there is failure intervention or recurrence of persistent arrhythmia, intervention is given again, but no more than three times in all.

### Statistical analysis

Continuous variables are expressed as median and interquartile range (25th and 75th percentile; in case of non-normal distribution) and compared by Mann–Whitney tests. Categorical variables are presented as frequencies or percentages and compared by *χ*^2^ or Fisher’s exact tests. Time to first LR was plotted using the Kaplan–Meier survival analysis and compared by the log-rank test. Factors with a *P-*value <0.05 on univariate analysis were considered in the multivariate model. Cox-regression analysis was performed to analyze the correlation between timing of first ER and risk of LR. Statistical tests and confidence intervals (CIs) with two-sided *P* < 0.05 were considered statistically significant. Statistical analysis was performed using the SPSS version 25.0 (IBM Inc., Armonk, NY, United States).

## Results

### Characteristics of patients

The flowchart of the present study is shown in Figure 1B. Out of 348 patients undergoing first ablation for persistent and long-standing persistent AF, 5 patients who failed to convert to SR were excluded after CA. A total of 343 patients (mean age 62.8 ± 9.0 years, male 63.3%) were included and followed up finally for a median of 13 months (inter-quartile range (IQR) 6–23 months) after CA. Long-standing persistent AF was noted in 237 (69.1%) patients. During the blanking period, 110 (32.1%) patients experienced ER with 78 (71.0%) and 16 (14.5%) patients as AF and AFL, respectively, and 16 (14.5%) patients as both AF and AFL. According to the occurrence of LR in patients with and without ER, differences in baseline characteristics are summarized in Table 1.

**Table 1:** Baseline characteristics of the study population according to the occurrence of LR in patients with and without ER.

	ER (+)			ER (−)		
	LR (+) (102)	LR (−) (8)	*P*	LR (+) (4)	LR (−) (229)	*P*
Age (year)	64 (58–69)	58 (48–65)	0.509	57 (62–71)	64 (57–70)	0.770
Male sex, *n* (%)	59 (60.8)	10 (76.9)	0.260	3 (60.3)	145 (63.6)	0.869
AF duration (months)	36 (16–111)	12 (11–54)	0.058	32 (24–55)	12 (2–24)	0.042
History of AFL, *n* (%)	6 (6.2)	1 (7.7)	0.839	1 (20.0)	19 (8.3)	0.357
CHA2DS2-VASc	2 (1–3)	2 (1–3)	0.821	1.5 (0.25–2)	2 (1–3)	0.277
Hypertension, *n* (%)	52 (53.6)	10 (76.9)	0.111	3 (60.0)	126 (55.3)	0.833
Diabetes mellitus, *n* (%)	22 (22.7)	5 (38.5)	0.214	0 (0.0)	33 (14.5)	0.359
Coronary artery disease, *n* (%)	25 (25.8)	4 (30.8)	0.741	2 (40.0)	48 (21.1)	0.307
Heart failure, *n* (%)	25 (25.8)	1 (7.7)	0.150	0 (0.0)	52 (22.8)	0.226
Chronic obstructive pulmonary disease, *n* (%)	4 (4.1)	0 (0.0)	0.456	0 (0.0)	7 (3.1)	0.579
History of ischemic stroke, *n* (%)	15 (15.5)	1 (7.7)	0.688	0 (0.0)	23 (10.1)	0.454
BMI (kg/m^2^)	20.7 (19.8–21.4)	21.5 (20.5–21.9)	0.195	20.5 (20.1–20.9)	20.7 (20.0–21.6)	0.519
LAA flow velocity (cm/s)	32 (25–38)	31 (24–47)	0.575	34.5 (26.3–57)	35 (26–47)	0.976
LA diameter (mm)	48 (44–50)	43 (39–45)	0.014	47 (45–49)	43 (40–46)	0.064
LV diameter (mm)	49 (44–52)	49 (47.5–52)	0.756	50 (40–52)	46 (43–50)	0.655
LV ejection fraction (%)	60 (55.5–64)	60 (57–63.5)	0.802	59 (51–68)	62 (56–66)	0.727
Albumin (g/L)	40.5 (38.4–42.17)	40.3 (39.0–41.7)	0.886	42.56 (38.52–45.36)	40 (38–43)	0.201
AST (µ/L)	22 (18.5–26)	20 (16–23)	0.041	28 (17–31)	23 (19–30)	0.335
ALT (µ/L)	20 (16–30)	25 (13–34.5)	0.985	28 (21–42)	22 (16–32)	0.149
Fasting blood Glucose (mmol/L)	5.53 (4.84–6.62)	6.16 (5.36–6.76)	0.357	5.44 (4.54–6.60)	5.4 (4.9–6.1)	0.997
Blood urea nitrogen (mmol/L)	6.12 (4.95–7.39)	5.85 (5.33–7.15)	0.708	5.47 (4.29–6.67)	6.0 (5.1–7.1)	0.673
Creatinine (mg/dl)	67 (58.5–78.5)	63 (61–84)	0.653	72 (61–77)	67 (59–75)	0.835
Uric acid (mmol/L)	373 (319.5–430.5)	362.0 (309.5–387.5)	0.287	360 (253–414)	377 (314–429)	0.446
TC (mmol/L)	4.45 (3.79–5.22)	4.22 (3.22–5.22)	0.993	4.44 (3.47–5.77)	4.5 (3.7–5.4)	0.794
Triglyceride (mmol/L)	1.02 (0.83–1.5)	1.19 (0.83–1.43)	0.637	1.37 (0.80–2.52)	1.1 (0.8–1.5)	0.409
HDL-c (mmol/L)	1.2 (1.03–1.42)	1.13 (0.94–1.23)	0.125	1.33 (1.11–1.63)	1.3 (1.1–1.5)	0.308
LDL-c (mmol/L)	2.87 (2.34–3.49)	2.73 (1.95–3.60)	0.663	2.90 (1.97–3.64)	2.7 (2.2–3.4)	0.844
HCY (mmol/L)	12.8 (11.0–15.4)	14.5 (10.7–16.1)	0.277	12 (10.9–17.8)	13.0 (10.8–15.2)	0.720
BNP (pg/ml)	156.9 (88.2–27 7.8)	161.1 (64.1–293.1)	0.594	175.7 (159.1–345.2)	162 (97–329)	0.423
D-Dimer (mg/L)	0.58 (0.46–0.82)	0.78 (0.53–0.93)	0.402	0.57 (0.50–0.69)	0.6 (0.5–0.8)	0.899
Hemoglobin (g/L)	148 (136.5–158)	152 (141–159.5)	0.362	144 (135.5–155.5)	149 (140–160)	0.427
Platelet (×10^9^/L)	206 (179–249.5)	246 (185–274.5)	0.313	194 (173.5–239.5)	209 (176–246)	0.551
WBC (×10^9^/L)	6.57 (5.19–7.87)	5.93 (4.73–7.35)	0.372	5.07 (4.57–5.50)	6.3 (5.3–7.4)	0.031
Substrate modification, *n* (%)	80 (82.5)	12 (92.3)	0.368	4 (80.0)	132 (57.9)	0.321
Anterior line ablation, *n* (%)	6 (6.2)	0 (0.0)	0.356	0 (0.0)	7 (3.1)	0.691
Roof line ablation, *n* (%)	57 (58.8)	6 (46.2)	0.388	1 (20.0)	78 (34.2)	0.507
Peri-mitral isthmus line ablation, *n* (%)	11 (11.3)	2 (15.4)	0.650	0 (0.0)	11 (4.8)	0.484
Ethanol infusion for Marshall bundle, *n* (%)	0 (0.0)	1 (7.7)	0.118	0 (0.0)	0 (0.0)	0.651
Peri-mitral isthmus line ablation, *n* (%)	17 (17.5)	4 (30.8)	0.268	1 (20.0)	68 (28.9)	0.662
CFAE-guided ablation, *n* (%)	37 (38.1)	3 (23.1)	0.368	2 (40.0)	45 (19.7)	0.265
Termination to SR by CA, *n* (%)	4 (4.1)	1 (7.7)	0.473	0 (0.0)	23 (10.1)	0.454
Termination to AFL by CA, *n* (%)	7 (7.2)	2 (15.4)	0.288	1 (20.0)	30 (13.2)	0.656
Termination to SR by Ibutilide, *n* (%)	23 (23.7)	5 (38.5)	0.252	3 (60.0)	113 (49.6)	0.644
Termination to SR by Ibutilide combined cardioversion, *n* (%)	21 (21.6)	1 (7.7)	0.459	0 (0.0)	24 (10.5)	0.444

AF, atrial fibrillation; AFL, atrial flutter; CFAE, complex fractionated atrial electrogram; CHA2DS2-VASc, congestive heart failure, hypertension, age, diabetes, previous stroke/transient ischemic attack, vascular disease, female sex; ER, early recurrence; LA, left atrium; LAA, left atrial appendage; LV, left ventricular; SR, sinus rhythm; BNP, B-type natriuretic peptide; TC, total cholesterol; LDL-c, low-density lipoprotein cholesterol; HDL-c, high-density lipoprotein cholesterol; ALT, alanine transaminase; AST, aspartate transaminase; WBC, white blood cell count; LR, late recurrence; CA, catheter ablation; BMI, body mass index; HCY, homocysteine.

Values are mean ± SD or %.

### Characteristics of ER of persistent AF

**Figure 2:**
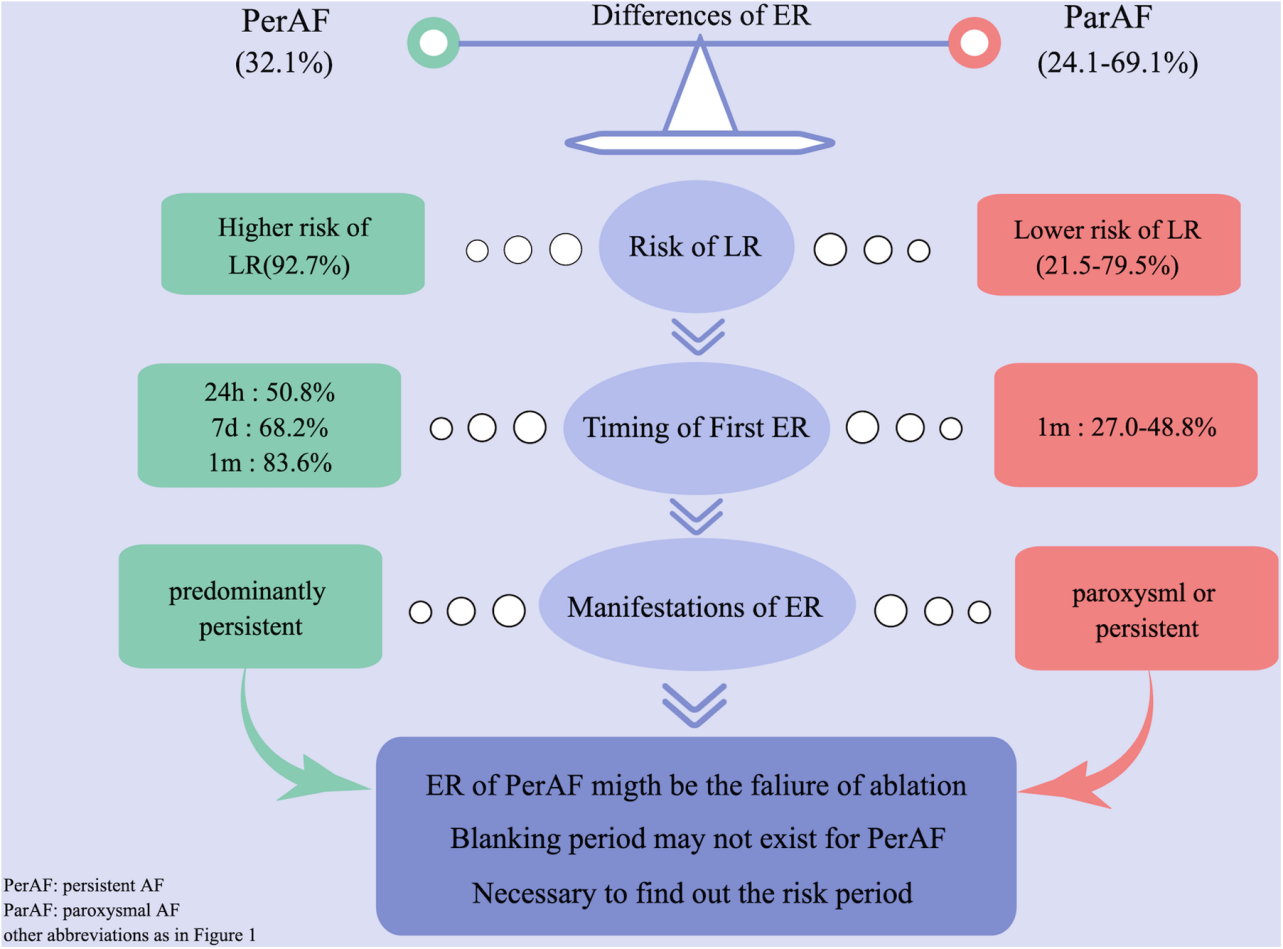
Difference of ER between persistent and paroxysmal AF by comparing with previous studies. AF, atrial fibrillation; ER, early recurrence.

**Figure 3:**
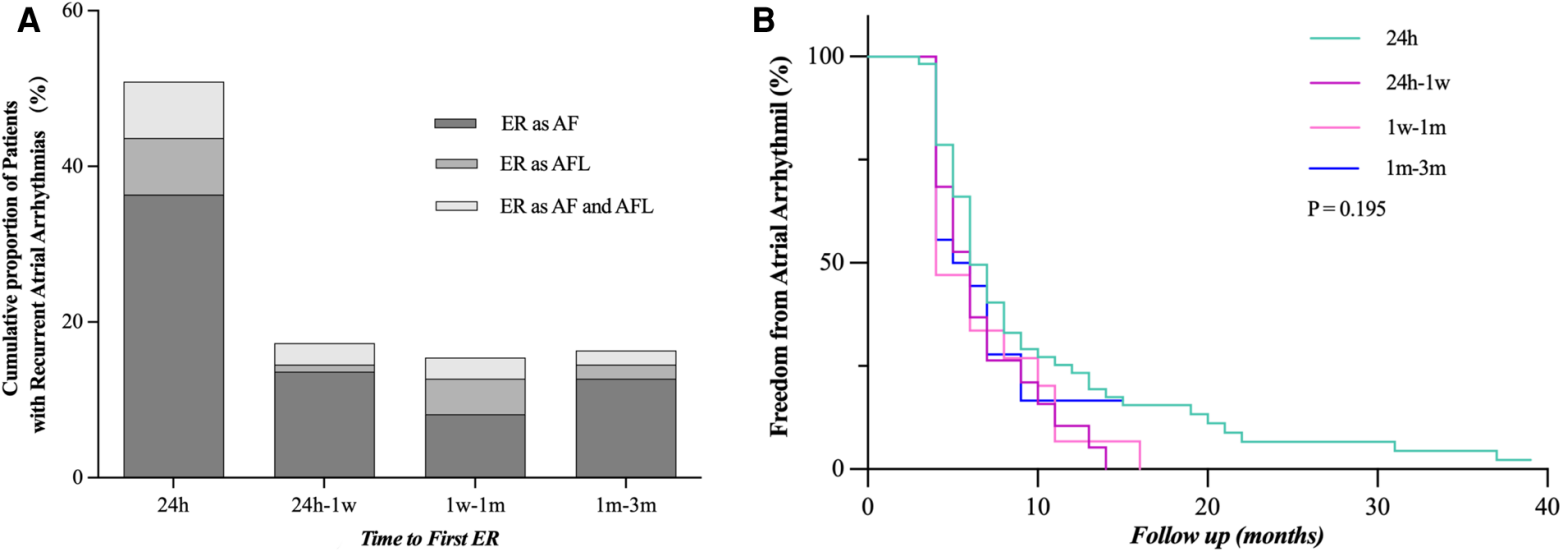
Time to the first episode of ER after catheter ablation. (**A**) Proportion of patients who developed ER after catheter ablation and time to recurrence are shown. (**B**) LR rate was not statistically different when compared between the groups distributed according to the timing of the first episode of ER (24 h, 24 h–1 week, 1 week–1 month, and 1–3 months, *P* = 0.195). ER, early recurrence, AF, atrial fibrillation, AFL, atrial flutter; LR, late recurrence.

We compared our results with previous studies on ER and found the following different characteristics of persistent AF (Figure 2). (1) First ER episode occurred in 50.9% of patients within 24 h and in 68.2% of patients within 7 days (Figure 3A). (2) ER is usually in the form of persistent atrial arrhythmia (89.1%). (3) LR was not related to timing of first ER manifestation events (*P* = 0.195) (Figure 3B).

### Risk factors for ER

Baseline characteristics of patients with and without ER are described in Table 2. Patients with ER had a longer mean AF duration [36 (IQR 12–108) vs. 12 (IQR 2–24) months, *P* < 0.001], more diabetes mellitus (24.5% vs. 14.2%, *P* = 0.018), a larger left atrial (LA) size [47.0 mm (IQR 43.0–50.0) vs. 43.2 mm (IQR 40.0–46.5), *P* < 0.001], a larger left ventricular (LV) diameter [49.0 mm (IQR 44.0–52.0) vs. 46.0 mm (IQR 43.0–50.0), *P* = 0.005], a lower left ventricular ejection fraction (LVEF) [60% (IQR 56–64) vs. 62% (IQR 56–66), *P* = 0.029], a lower left atrial appendage (LAA) flow velocity [32 cm/s (IQR 25–40) vs. 35 cm/s (IQR 27–46), *P* = 0.033], a lower HDL-c [1.18 mmol/L (IQR 1.03–1.36) vs. 1.25 mmol/L (IQR 1.07, 1.47), *P* = 0.032], more additional substrate modification (83.6% vs. 58.4%, *P* < 0.001), roof line ablation (57.3% vs. 33.9%, *P* < 0.001), peri-mitral isthmus line ablation (11.8% vs. 4.7%, *P* < 0.001), CFAE ablation (36.4% vs. 20.2%, *P* < 0.001), termination to SR by Ibutilide (25.5% vs. 49.8%, *P* < 0.001), and termination to SR by Ibutilide combined cardioversion (20.0% vs. 10.3%, *P* = 0.014).

**Table 2:** Baseline characteristics and procedural data of patients with and without early recurrence.

	ER (+) (110)	ER (−) (233)	*P*-value
Age (years)	64 (57.68)	63 (57–70)	0.619
Male sex (%)	69 (62.7)	148 (63.5)	0.490
AF duration (months)	36 (12–108)	12 (2–24)	<0.001
History of AFL, *n* (%)	7 (6.4)	20 (8.6)	0.316
CHA2DS2-VASc	2 (1–3)	2 (1–3)	0.222
Hypertension, *n* (%)	62 (56.4)	129 (55.4)	0.478
Diabetes mellitus, *n* (%)	27 (24.5)	33 (14.2)	0.018
Coronary artery disease, *n* (%)	29 (26.4)	50 (21.5)	0.192
Heart failure, *n* (%)	26 (23.6)	52 (22.3)	0.786
Chronic obstructive pulmonary disease, *n* (%)	4 (3.6)	7 (3.0)	0.492
History of ischemic stroke, *n* (%)	16 (14.5)	23 (9.9)	0.138
BMI (kg/m^2^)	20.8 (19.8–21.5)	20.8 (20.0–21.7)	0.626
LAA flow velocity (cm/s)	32 (25–40)	35 (27–46)	0.033
LA diameter (mm)	47 (43–50)	43.2 (40–46.5)	<0.001
LV diameter (mm)	49 (44–52)	46 (43–50)	0.005
LV ejection fraction (%)	60 (56–64)	62 (56–66)	0.029
Blood tests			
Albumin (g/L)	40.45 (38.46–42.13)	40.3 (38.0–42.7)	0.921
AST (µ/L)	21.5 (18–26)	23 (19–30)	0.052
ALT (µ/L)	20 (16–30.25)	22 (16–22)	0.323
Fasting blood glucose (mmol/L)	5.60 (4.91–6.67)	5.42 (4.91–6.12)	0.163
Blood urea nitrogen (mmol/L)	6.09 (5.10–7.33)	6.04 (5.08–7.02)	0.738
Creatinine (mg/dl)	67 (59–79)	67 (59–75)	0.767
Uric acid (mmol/L)	370.5 (322.3–421.8)	376 (314–428)	0.993
TC (mmol/L0	4.45 (3.79–5.27)	4.52 (3.75–5.39)	0.954
Triglyceride (mmol/L)	1.05 (5.83–1.49)	1.08 (0.79–1.49)	0.788
HDL-c (mmol/L)	1.18 (1.03–1.36)	1.25 (1.07–1.47)	0.032
LDL-c (mmol/L)	2.80 (2.22–3.48)	2.75 (2.19–3.40)	0.772
HCY	13 (10.98–15.43)	13.0 (10.80–15.15)	0.817
BNP (pg/ml)	160.13 (82.1–274.5)	165.21 (98.85–329.21)	0.161
D-Dimer (mg/L)	0.59 (0.47–0.85)	0.57 (0.46–0.79)	0.570
Hemoglobin (g/L)	148 (138–158)	149 (140–160)	0.372
Platelet (×10^9^/L)	210 (180–252)	207.0 (176.5–246.0)	0.485
WBC (×10^9^/L)	6.52 (5.11–7.73)	6.28 (5.24–4.72)	0.529
Substrate modification, *n* (%)	92 (83.6)	136 (58.4)	<0.001
Anterior line ablation, *n* (%)	6 (5.5)	7 (3.0)	0.207
Roof line ablation, *n* (%)	63 (57.3)	79 (33.9)	<0.001
Peri-mitral isthmus line ablation, *n* (%)	13 (11.8)	11 (4.7)	0.016
Ethanol infusion for Marshall bundle, *n* (%)	1 (0.9)	0 (0.0)	0.145
Peri-tricuspid isthmus line ablation, *n* (%)	21 (19.1)	67 (28.8)	0.056
CFAE-guided ablation, *n* (%)	40 (36.4)	47 (20.2)	0.001
Termination to SR by CA, *n* (%)	5 (4.5)	23 (9.9)	0.093
Termination to AFL by CA, *n* (%)	9 (8.2)	31 (13.3)	0.168
Termination to SR by Ibutilide, *n* (%)	28 (25.5)	116 (49.8)	<0.001
Termination to SR by Ibutilide combined cardioversion, *n* (%)	73 (66.4)	78 (33.5)	<0.001

AF, atrial fibrillation; AFL, atrial flutter; CFAE, complex fractionated atrial electrogram; CHA2DS2-VASc, congestive heart failure, hypertension, age, diabetes, previous stroke/transient ischemic attack, vascular disease, female sex; ER, early recurrence; LA, left atrium; LAA, left atrial appendage; LV, left ventricular; SR, sinus rhythm; TC, total cholesterol; LDL-c, low-density lipoprotein cholesterol; HDL-c, high-density lipoprotein cholesterol; BNP, B-type natriuretic peptide; ALT, alanine transaminase; AST, aspartate transaminase; WBC, white blood cell count; CA, catheter ablation.

Values are mean ± SD or %.

Multivariate analysis revealed that AF duration (OR 1.006, 95% CI 1.004–1.008, *P* < 0.001), LA size (OR 1.063, 95% CI 1.018–1.110, *P* = 0.006), substrate modification (OR 2.34, 95% CI 1.237–4.427, *P* = 0.009), and termination to SR by Ibutilide combined cardioversion (OR 3.945, 95% CI 1.875–8.3, *P* < 0.001) were independent risk factors for ER (Table 3).

**Table 3:** Risk factors for early recurrence.

	OR	95% CI for OR	*P*-value
AF duration	1.006	1.004–1.008	<0.001
Diabetes mellitus	2.083	1.305–3.325	0.102
LAA flow velocity	0.990	0.973–1.007	0.232
LA diameter	1.063	1.018–1.110	0.006
LV diameter	0.990	0.953–1.029	0.619
LV ejection fraction	0.978	0.955–1.002	0.069
HDL-c	0.59	0.278–1.254	0.170
Substrate modification	2.340	1.237–4.427	0.009
Roof line ablation	0.955	0.602–1.516	0.845
Peri-mitral isthmus line ablation	1.150	0.600–2.201	0.674
CFAE-guided ablation	0.970	0.604–1.557	0.899
Termination to SR by Ibutilide,	1.393	0.636–3.051	0.407
Termination to SR by Ibutilide combined cardioversion	3.945	1.875–8.300	<0.001

CI, confidence interval; AF, atrial fibrillation; LA, left atrium; LAA, left atrial appendage; LV, left ventricular; CFAE, complex fractionated atrial electrogram; HDL-c, high-density lipoprotein cholesterol; SR, sinus rhythm.

Hazard ratios are calculated by multivariate Cox-regression analysis.

### Risk factors for LR

During the follow-up, LR occurred in 106 (30.9%) patients. On univariate analysis, patients with LR had a longer mean AF duration [36 months (IQR 19–108) vs. 12 months (IQR 2–24); *P* < 0.001], larger LA size [47 mm (IQR 44–50) vs. 43 (IQR 40–46); *P* < 0.001], a lower LVEF [60% (IQR 56–64) vs. 62% (IQR 57–66); *P* = 0.024], a larger left ventricular diameter [49 mm (IQR 44–52) vs. 46 mm (IQR 43–50); *P* = 0.005], a lower LAA flow velocity [32 cm/s (IQR 25–38) vs. 35 cm/s (IQR 26–47); *P* = 0.016], more substrate modification (83% vs. 59.1%, *P* < 0.001), roof line ablation (55.7% vs. 35.0%, *P* < 0.001), peri-mitral isthmus line ablation (12.3% vs. 4.6%, *P* = 0.012), peri-tricuspid isthmus line ablation (17.9% vs.29.1%, *P* = 0.018), CFAE-guided ablation (38.7% vs. 19.4%, *P* < 0.001), termination to SR by Ibutilide (29.1% vs. 17.9%, *P* = 0.018), and termination to SR by Ibutilide combined cardioversion (38.7% vs. 19.4%, *P* < 0.001) compared to patients without LR (Table 4).

**Table 4:** Baseline characteristics and procedural data of patients with and without late recurrence.

	LR (+) (106)	LR (−) (237)	*P*-value
ER (%)	102 (96.2)	8 (3.4)	<0.001
Age (years)	64 (58–68)	64 (57–69)	0.990
Male sex, *n* (%)	64 (60.4)	153 (64.6)	0.267
AF duration (months)	36 (19–108)	12 (2–24)	<0.001
History of AFL, *n* (%)	7 (6.6)	20 (8.4)	0.365
CHA2DS2-VASc	2 (1–3)	2 (1–3)	0.286
Hypertension, *n* (%)	58 (54.7)	133 (56.1)	0.450
Diabetes mellitus, *n* (%)	25 (23.6)	35 (14.8)	0.035
Coronary artery disease, *n* (%)	31 (29.2)	48 (20.3)	0.047
Heart failure, *n* (%)	26 (24.5)	51 (21.5)	0.314
Chronic obstructive pulmonary disease, *n* (%)	4 (3.8)	7 (3.0)	0.457
History of ischemic stroke (%)	15 (14.2)	24 (10.1)	0.183
BMI (kg/m^2^)	20.8 (19.8–21.6)	20.7 (20.0–21.7)	0.626
LAA flow velocity (cm/s)	32 (25–38)	35 (26–47)	0.016
LA diameter (mm)	47 (44–50)	43 (40–46)	<0.001
LV diameter (mm)	49 (44–52)	46 (43–50)	0.005
LV ejection fraction (%)	60 (56–64)	62 (57–66)	0.024
Albumin (g/L)	40.5 (38.4–42.2)	40.2 (38.0–42.7)	0.979
AST (µ/L)	22 (18–26)	23 (19–30)	0.056
ALT (µ/L)	20 (16.30)	22 (16–33)	0.204
Fasting blood glucose (mmol/L)	5.53 (4.81–6.68)	5.42 (4.92–6.17)	0.379
Blood urea nitrogen (mmol/L)	6.13 (4.91–7.33)	6.03 (5.10–7.02)	0.792
Creatinine (mg/dl)	67 (59–77)	67 (59–75)	0.894
Uric acid (mmol/L)	368 (313–422)	374 (316–428)	0.764
TC (mmol/L)	4.45 (3.81–5–23)	4.52 (3.74–5.40)	0.883
Triglyceride (mmol/L)	1.05 (0.84–1.53)	1.07 (0.79–1.48)	0.936
HDL-c (mmol/L)	1.19 (1.03–1.41)	1.24 (1.03–1.46)	0.484
LDL-c (mmol/L)	2.81 (2.24–3.84)	2.75 (2.18–3.41)	0.652
HCY (mmol/L)	12.9 (11.0–15.3)	13.0 (10.8–15.3)	0.925
BNP (pg/ml)	160.1 (85.8–266.1)	165.2 (97.4–329.7)	0.147
D-Dimer (mg/L)	0.58 (0.47–0.83)	0.57 (0.46–0.79)	0.210
Hemoglobin (g/L)	148 (139–158)	149 (140–160)	0.188
Platelet (×10^9^/L)	205.5 (180.0–215.5)	210.0 (176.5–247.5)	0.792
WBC (×10^9^/L)	6.41 (5.12–7.71)	6.34 (5.29–7.48)	0.971
Substrate modification, *n* (%)	88 (83.0)	140 (59.1)	<0.001
Anterior line ablation, *n* (%)	6 (5.7)	7 (3.0)	0.180
Roof line ablation, *n* (%)	59 (55.7)	83 (35.0)	<0.001
Peri-mitral isthmus line ablation, *n* (%)	13 (12.3)	11 (4.6)	0.012
Ethanol infusion for Marshall bundle, *n* (%)	0 (0)	1 (0.4)	0.515
Peri-tricuspid isthmus line ablation, *n* (%)	69 (29.1)	19 (17.9)	0.018
CFAE-guided ablation, *n* (%)	41 (38.7)	46 (19.4)	<0.001
Termination to SR by CA, *n* (%)	5 (4.7)	23 (9.7)	0.085
Termination to AFL by CA, *n* (%)	8 (7.5)	32 (13.5)	0.077
Termination to SR by Ibutilide, *n* (%)	26 (24.5)	118 (49.8)	<0.001
Termination to SR by Ibutilide combined cardioversion, *n* (%)	72 (67.9)	79 (33.3)	<0.001

ER, early recurrence; AF, atrial fibrillation; AFL, atrial flutter; CHA2DS2-VASc, congestive heart failure, hypertension, age, diabetes, previous stroke/transient ischemic attack, vascular disease, female sex; LA, left atrium; LAA, left atrial appendage; LV, left ventricular; ALT, alanine transaminase; AST, aspartate transaminase; TC, total cholesterol; LDL-c, low-density lipoprotein cholesterol; HDL-c, high-density lipoprotein cholesterol; BNP, B-type natriuretic peptide; WBC, white blood cell count; CFAE, complex fractionated atrial electrogram; SR, sinus rhythm; CA, catheter ablation.

Values are mean ± SD or %.

In multivariate analysis, ER (OR 120.505, 95% CI 41.517–349.77, *P* < 0.001), LA size (OR 1.063, 95% CI 1.015–1.112, *P* = 0.009), and termination to SR by Ibutilide combined cardioversion (OR 2.347, 95% CI 1.042–5.288, *P* = 0.039) were the independent predictors of LR (Table 5). ER was observed at a significantly higher rate in patients with LR (96.2% vs. 3.4%, *P* < 0.001) than in patients without LR (Table 5).

**Table 5:** Risk factors for late recurrence.

	OR	95% CI for OR	*P-*value
ER	120.505	41.517–349.77	<0.001
AF duration	1.001	0.998–1.003	0.596
Coronary artery disease	1.287	0.814–2.035	0.280
LAA flow velocity	0.999	0.979–1.018	0.886
LA diameter	1.063	1.015–1.112	0.009
LV diameter	0.979	0.936–1.025	0.375
LV ejection fraction	0.996	0.971–1.022	0.747
Substrate modification	0.925	0.459–1.864	0.827
Roof line ablation	0.672	0.411–1.097	0.112
Peri-mitral isthmus line ablation	1.403	0.748–2.633	0.291
Peri-tricuspid isthmus line ablation	0.668	0.353–1.264	0.215
CFAE-guided ablation	1.031	0.633–1.678	0.904
Termination to SR by Ibutilide	1.513	0.629–3.637	0.355
Termination to SR by Ibutilide combined cardioversion	2.347	1.042–5.288	0.039

CI, confidence interval; ER, early recurrence; AF, atrial fibrillation; LA, left atrium; LAA, left atrial appendage; LV, left ventricular; CFAE, complex fractionated atrial electrogram; SR, sinus rhythm.

Hazard ratios are calculated by multivariate Cox-regression analysis.

### Relationship between ER and LR

**Figure 4:**
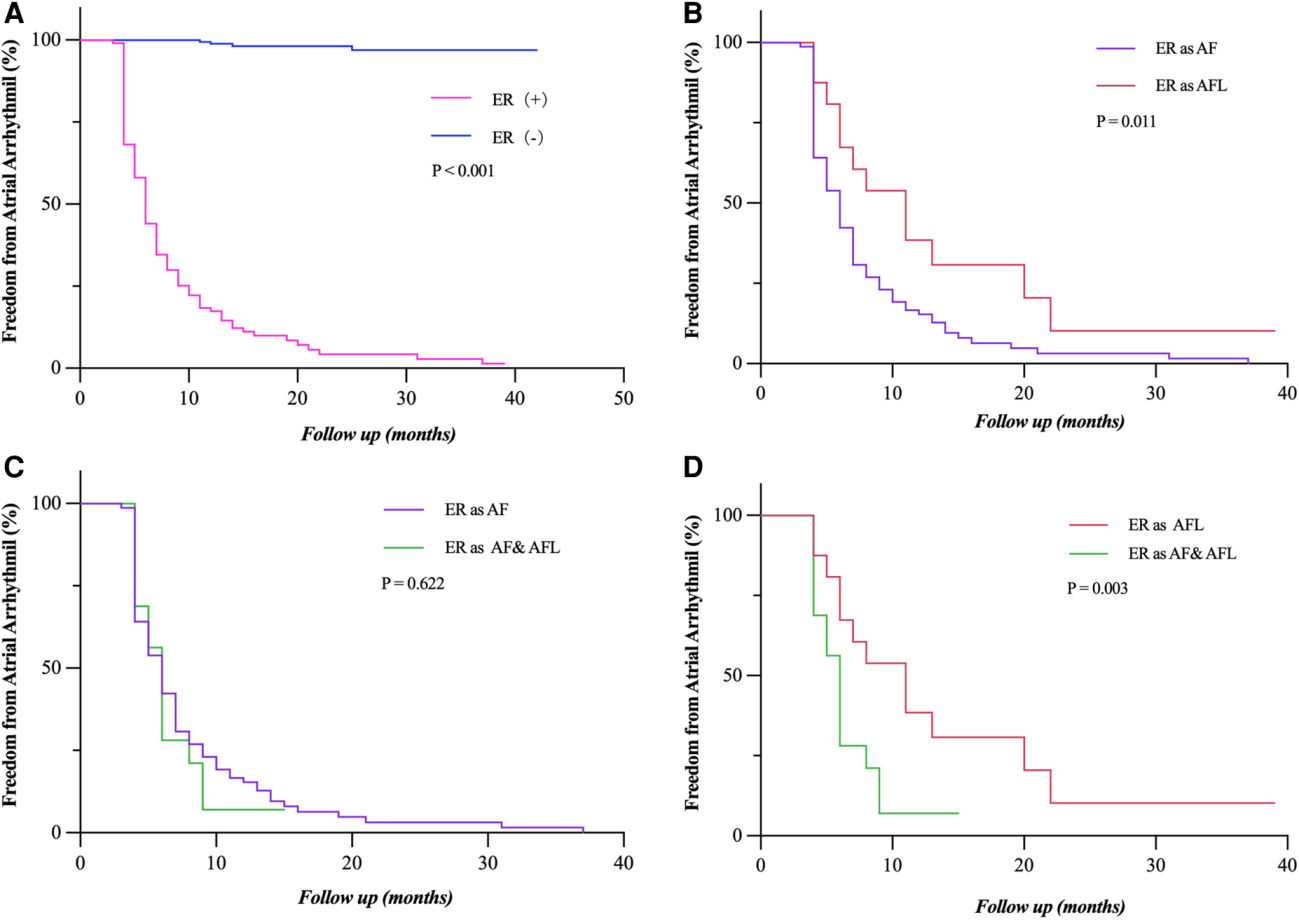
Freedom from atrial tachyarrhythmia recurrence. (**A**) Patients who experienced ER (as AF, AFL, and both AF and FL) had a significantly lower rate of freedom from LR (*P* < 0.001). (**B,D**) Patients who had ER as AF and both AF and AFL showed a significantly lower rate of freedom from LR compared to those with ER as AFL (*P* = 0.011; *P* = 0.003). (**C**) The rate of freedom from LR had no statistical difference between the patients who had ER as AF and as both AF and AFL (*P* = 0.622). Incidence curves were compared using the log-rank test. ER, early recurrence; LR, late recurrence; AF, atrial fibrillation; AFL, atrial flutter.

During long-term follow-up, the LR rate was significantly higher in patients with ER (92.7% vs. 1.7%, *P* < 0.001) than those without ER (Figure 4A). ER as AFL had a lower risk of LR compared with ER as AF and both AF and AFL (*P* = 0.011; *P* = 0.003) (Figures 4B,D). Compared with ER as AF (Figure 4C), ER as both AF and AFL had a similar risk of LR (*P* = 0.622). The relationship between the types of ER and LR can be further clarified by expanding the sample size.

### Relationship between early intervention and LR

**Figure 5:**
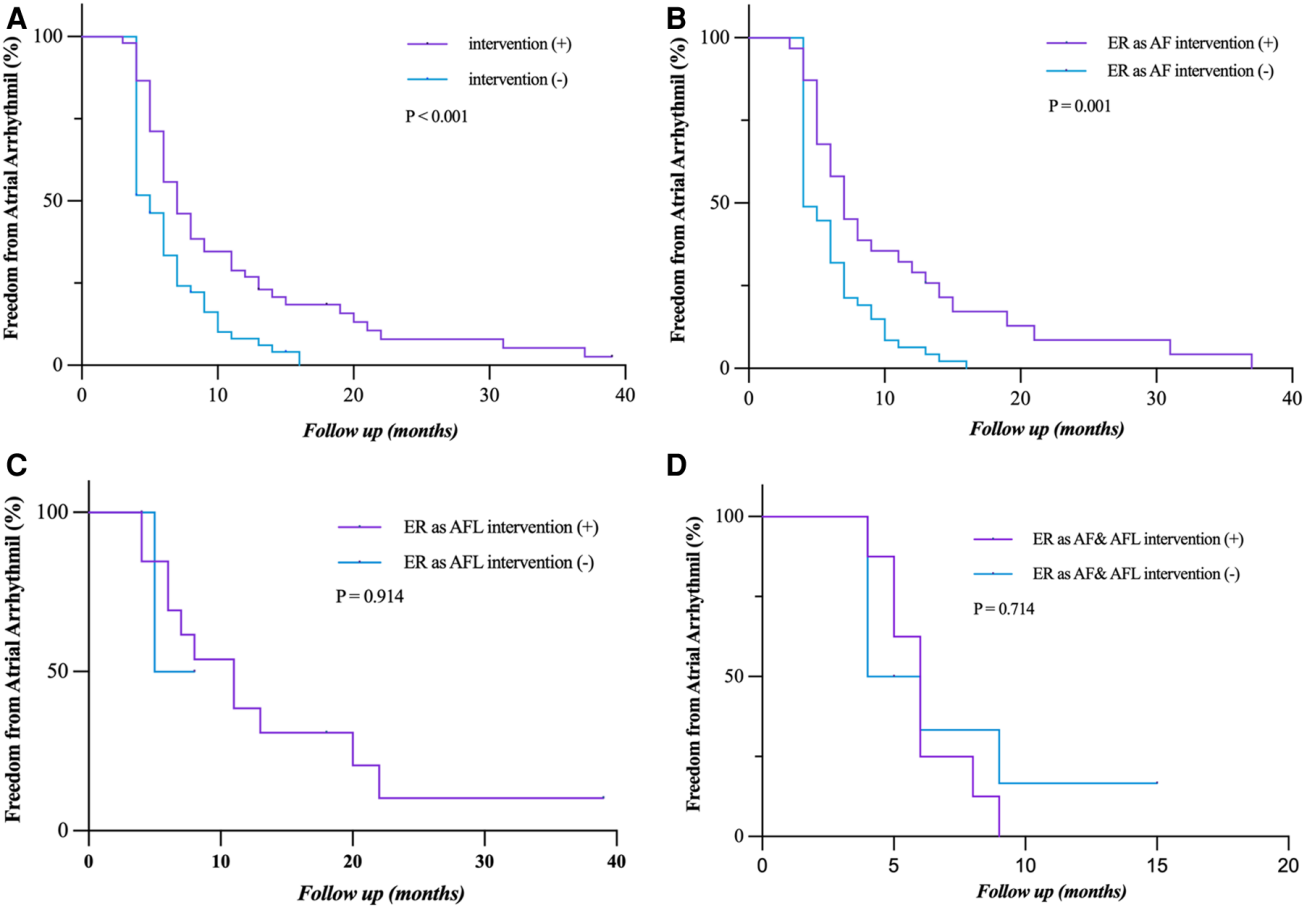
Freedom from atrial tachyarrhythmia recurrence after early intervention for ER. (**A,B**) Patients who had early intervention after ER (as AF, AFL, and both AF and AFL) had a significantly lower rate of freedom from LR (*P* = 0.001) in the short term, but not in the long term, which is more obvious in the patients who had ER as AF (*P* = 0.001). (**C,D**) The rate of freedom from LR had no statistical difference between the patients who had early intervention after ER as AFL and as both AF and AFL (*P* = 0.914, *P* = 0.714). Incidence curves were compared using the log-rank test. ER, early recurrence; AF, atrial fibrillation; AFL, atrial flutter; LR, late recurrence.

Early intervention was performed in 48 (47.3%) patients with persistent ER during the blanking period. Patients who had early intervention after ER (as AF, AFL, and both AF and AFL) had a significantly lower rate of freedom from LR (*P* = 0.001) in the short term, but not in the long term, which is more obvious in patients who had ER as AF (*P* = 0.001) (Figures 5A,B). The rate of freedom from LR had no statistical difference between the patients who had early intervention after ER as AFL and as both AF and AFL (*P* = 0.914; *P* = 0.714) (Figures 5C,D). The sample size of patients with ER as AFL and both AF and AFL is too small that cannot be accurately analyzed.

### Risk period for LR

**Figure 6:**
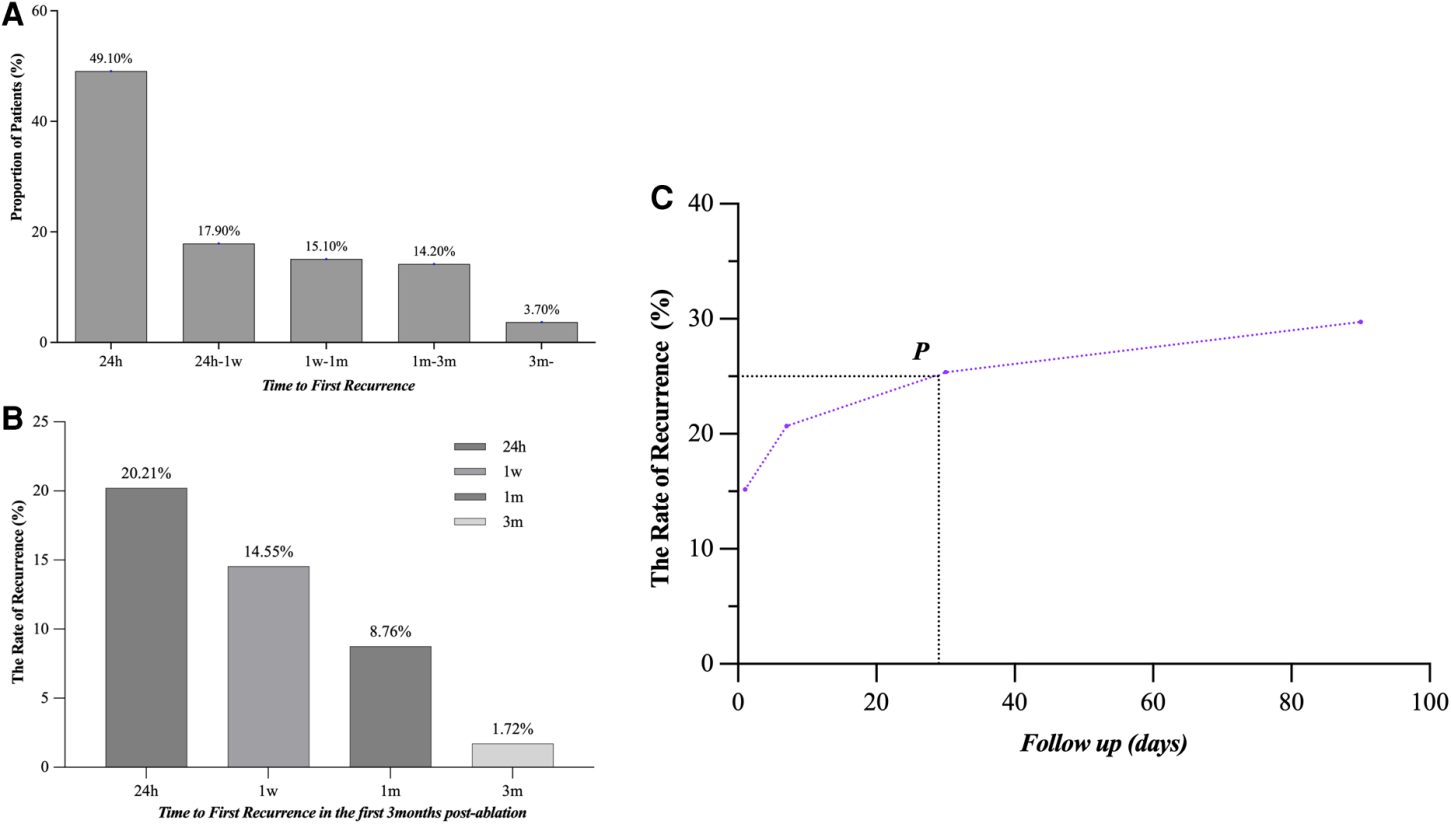
Time to the atrial tachyarrhythmia recurrence after CA. (**A**) The proportion was 49.1%, 17.9%, 15.1%, 14.2%, and 3.7%, respectively, in 24 h, 24 h–1 week, 1 week–1 month, 1–3 months, and after 3 months in AF patients with LR. (**B**) The LR probabilities of patients without recurrence in 24 h, 1 week, 1 month, and 3 months after CA were 20.21%, 14.55%, 8.76% and 1.72%, respectively. (**C**) The overall recurrence rate at 1 month after CA reached 25%, with 86.8% of these patients still recurring during the follow-up. LR, late recurrence; AF, atrial fibrillation; CA, catheter ablation.

In a subgroup analysis (LR, *n* = 106), we assume that there is no blanking period after CA. Recurrence of arrhythmia occurred in 71/106 (70.0%) patients during the first week and 92/106 (86.8%) patients within the first month (Figure 6A). The occurrence rate of LR was 35/343 (14.45%) and 22/251 (8.76%) in patients who did not experience recurrence in the first week and first month, respectively (Figure 6B). LR occurred within the first month in 25.4% of all patients (Figure 6C).

## Discussion

### Main findings

In the present study, we first summarized the characteristics and significances of ER after CA in patients with persistent AF (Figure 2). (1) Patients experiencing ER have a significantly higher risk (92.7%) of LR compared with those without ER. (2) The majority (50.9%) of first ER occurred in the first 24 h after CA. (3) ER was predominantly present as persistent. (4) LR was not related to the timing of the first ER. (5) ER as AFL had a lower risk of LR compared with ER as AF and as both AF and AFL. (6) Early intervention during blanking period could improve the prognosis in the short term, but could not prevent more LR. (7) Only 8.76% of patients who did not have recurrence within the first 1 month after CA had LR; we called this period the “risk period” of recurrence.

Our findings are not in accordance with the current guideline recommendation that ER is a common phenomenon after CA for AF.

### Characteristics of ER in patients with persistent AF

#### The majority of ER first occurred in the initial 24 h after CA

ER showed the highest incidence in the first week after CA. Nearly half of the patients had ER within the first 24 h, which was not consistent with the findings of previous studies (3, 10–13). The theoretical basis of the blanking period is related to transient inflammatory response, autonomic nervous system imbalance, and lesion maturation after CA. These studies do not explain the earlier recurrence in persistent AF, as is usually present after CA in paroxysmal AF. Another theory that ER is associated with pulmonary vein reconnection is also not supported in persistent AF (14). ER confined to the first 4 weeks after PVI is not associated with pulmonary vein reconnection. Patients with ER have a higher risk of LR after repeat CA, but pulmonary vein reconnection is less likely after repeat CA (15). Furthermore, AI-guided ablation may also reduce pulmonary vein reconnection. It is inferred that the presence of substrates may lead to earlier ER.

#### ER was predominantly persistent

ER was predominantly persistent, which is in striking contrast to previous reports of paroxysmal AF cohorts (10, 11, 16). In the early stage of AF, triggers from the pulmonary veins are the main mechanism. However, the more important mechanism in the persistence of AF is the change of underlying atrial substrate with remodeling in the later stage (17). We consider that the atrial substrate in those patients was too strong to be sufficiently modified by the ablation strategies applied in our study, and it may result in the recurrence of persistent atrial arrhythmias. Popa reported that 18% of patients of ER in persistent AF experienced spontaneous conversion to SR (18). This phenomenon was also confirmed in our study that it was observed in only 10.9% of patients with ER. Ibutilide combined cardioversion and larger LA were the independent predictors of LR, which indicated that the substrate of these patients may be stronger and may be a more important mechanism in the persistence of AF.

#### The timing of ER was not associated with the incidence of LR

We also found a different correlation between ER timing and LR than previously described for paroxysmal AF, where the risk of LR is known to gradually increase with later ER (3, 10, 19). In our cohort, the risk of LR was rather equally high regardless of the timing of ER.

The above characteristics confirm that the occurrence of ER may reflect the original AF substrate but is not a result of a solely transient mechanism. If so, this would support early re-ablation in these patients.

### Value of ER and LR

We found that the patients with ER are inevitably subject to LR. This adds to the growing body of evidence that ER is highly predictive of LR in paroxysmal AF (3, 19, 16). The type of LR (68.0% AF and 17.0% AFL, 15% both AF and AFL) mainly corresponded to the ER type (71.0% AF, 14.5% AFL, and 14.5% both AF and AFL), as reported previously (20). These data support an almost equal relationship between ER and LR in patients with persistent AF. Thus, we guess that the occurrence of ER might be a surrogate marker of the severity of only AF itself.

In our study, AF often transforms into AFL during the ablation of atrial substrate rather than PVI, and the patients with ER as AFL had a significantly lower risk of LR. That may indicate that ER as AFL might suggest a reduction in AF substrate after CA. Thus, the clinical significance and underlying pathophysiology of ER as AFL should be explored further.

Among LR patients, 86.8% occurred within 1 month after CA, and among patients without ER within 1 month, only 8.76% had LR, suggesting that 1 month after CA may be the “risk period” for LR, which has never been reported in previous studies.

### Intervention for ER

Early intervention is thought to promote reverse remodeling processes in patients with ER (21). However, previous studies have failed to reach a consistent conclusion as to whether intervention usage in patients with ER after CA was associated with the reduction of AF recurrence, and there are few studies on patients with persistent AF. Several studies with a majority of patients with persistent AF have found that early intervention after CA did not prevent more LR (22, 23). On the contrary, in other studies, intervention after CA could prevent LR in patients with paroxysmal AF (21, 24). Our results showed that intervention could not affect the outcome of patients with ER of persistent AF after CA, which is consistent with the former.

### CF and AI-guided ablation could improve durability of line isolation

Our study also reports the ER of AF ablated by the CF catheter and AI-guided ablation for the first time. With the introduction of CF-sensing catheters and AI algorithms to improve lesion quality or more transmural energy sources, we enhanced PVI and linear block of the additional ablation. In previous studies, it has been demonstrated that CF-sensing catheters could increase efficiency in the ablation of persistent AF caused by the reduction of PV reconnection (25–27). Successful complex electrogram ablation and the achievement of linear block minimized the occurrence of new arrhythmias (28). In this case, there is further evidence that the unablated substrates may be a more important mechanism for the recurrence of persistent AF.

## Outlook and clinical implications

In the present study, ER in persistent AF shows different characteristics and prognostic significance. We recommend that patients with persistent AF undergoing ER should be informed of their high risk for LR without waiting for the onset and end of ER. We initially proposed that there is no so-called “blanking period” of 3 months after CA for patients with persistent AF, but there is a “risk period” of 1 month after CA, which is in contrast with the guideline. For patients with ER of persistent AF after CA, early re-ablation may reduce LR.

## Limitations

The results from this retrospective single-center analysis are not free from the inherent limitations of this design. First, ER was not determined by implantable event recorder devices or trans-telephonic monitoring, which have not provided a more accurate time of ER, and as a result, the exact time of what we call the risk period is not obtained. Second, we did not study further whether or not early re-ablation vs. late re-ablation following an unsuccessful CA for persistent AF would provide any additional benefit or value. Third, many patients received non-PVI ablation, but we have not explored further whether non-PVI ablation was associated with ER. Fourth, although we concluded the difference of ER between paroxysmal and persistent AF by collecting previous studies on ER of paroxysmal AF, we did not conduct a controlled study of patients with persistent and paroxysmal AF in this study. Finally, the study consisted entirely of East Asian patients, and whether these results apply to other ethnic groups needs further investigation.

## Conclusions

For persistent AF, ER is a strong independent predictor of LR, which challenges the fact that ER is merely a transient phenomenon. In patients with persistent AF, most of the LR occurred in the first month after CA. Based on the above two points, we hypothesized that there may not be a so-called 3-month “blanking period” for patients with persistent AF, but there is a “risk period” of 1 month after CA, which can be tested in future studies.

## Data Availability

The original contributions presented in the study are included in the article, further inquiries can be directed to the corresponding authors.
